# Comments on Public Health Aspects of Paediatric Dental Treatment under General Anaesthesia. *Dent. J.* 2016, *4*, 20

**DOI:** 10.3390/dj4030023

**Published:** 2016-07-19

**Authors:** Ziad D. Baghdadi

**Affiliations:** Department of Community Health & Epidemiology, University of Saskatchewan College of Medicine, Saskatoon, SK S7N 1L7, Canada; ziad.baghdadi@usask.ca; Tel.: +1-306-880-8843

I would like to comment on an article by Thomson [1] recently published in *Dentistry Journal*. First of all, I thank the author for his excellent and timely review. However, I would like to add some notes to complete the review. In the section titled, “A Research Agenda—What Else Do We Need to Know?” Thomson [1] stated that there are a number of areas where more research is required. Particularly, he noted that there is a need to assess test-retest reliability of the OHRQoL instruments. In fact, the work cited by the author (Reference [33]) by Baghdadi [2] assessed test-retest reliability of the P-CPQ and FIS; intraclass correlation coefficients were 0.93 and 0.84 for 16-item P-CPQ and 8-item FIS respectively, indicating an excellent agreement with repeated administration of the scales. Thomson [1] also raised the issue of sustainability of the effects of DGA on OHRQoL: How long do they last? In fact, at least two studies reported on this: the first one by Baghdadi [3] who reported moderate to large effects in mid-term follow-up (6–9 months) and the second by El-Meligy et al. [4] who reported a significant long-term effect on children’s OHRQoL extending up to 12 months postoperatively. It is worth mentioning that the latter study [4] involved children with special health care needs. Baghdadi is currently analysing data on long-term effects of DGA on children’s OHRQoL. In addition, Thomson [1] noted the need to look at the nature of the treatment rendered under GA: Restorative versus exodontic. Wong et al. [5] assessed the changes in OHRQoL in children requiring dental extractions under GA and reported large effect sizes two-week post extraction. Finally, Thomson [1] suggested that characteristics such as personality and other family functioning could affect how to rate OHRQoL. Actually, there are some studies that examined the influence of psychological characteristics and other related factors on perceived OHRQoL. Agou et al. (2011) [6] found that children with better psychological well-being were more likely to report better OHRQoL regardless of their orthodontic treatment status. In another study, Agou et al. (2008) [7] found that self-esteem is a more salient determinant of OHRQoL in children seeking orthodontic treatment. Kumar et al. [8] systematically reviewed 36 articles reporting on the impact of parental socio-economic status and home environment characteristics on children’s OHRQoL. Baghdadi and Muhajrine [9] demonstrated that some parental characteristics, such as education and gender, have an effect on their children’s OHRQoL after dental treatment under GA. Nevertheless, I concur that more studies are warranted taking the points raised by Thomson into consideration. Lastly, I appreciate the major contributions of Thomson over the years to the field of pediatric dentistry.
